# Butanol production in *S. cerevisiae* via a synthetic ABE pathway is enhanced by specific metabolic engineering and butanol resistance

**DOI:** 10.1186/s13068-015-0281-4

**Published:** 2015-07-08

**Authors:** R. Swidah, H. Wang, P.J. Reid, H.Z. Ahmed, A.M. Pisanelli, K.C. Persaud, C.M. Grant, M.P. Ashe

**Affiliations:** The Faculty of Life Sciences, The Michael Smith Building, The University of Manchester, Oxford Rd., Manchester, M13 9PT UK; School of Chemical engineering and Analytical Science, The Mill, The University of Manchester, Sackville St., Manchester, M139PL UK

**Keywords:** Biobutanol, *Saccharomyces cerevisiae*, ABE pathway

## Abstract

**Background:**

The fermentation of sugars to alcohols by microbial systems underpins many biofuel initiatives. Short chain alcohols, like n-butanol, isobutanol and isopropanol, offer significant advantages over ethanol in terms of fuel attributes. However, production of ethanol from resistant *Saccharomyces cerevisiae* strains is significantly less complicated than for these alternative alcohols.

**Results:**

In this study, we have transplanted an n-butanol synthesis pathway largely from *Clostridial* sp. to the genome of an *S. cerevisiae* strain. Production of n-butanol is only observed when additional genetic manipulations are made to restore any redox imbalance and to drive acetyl-CoA production. We have used this butanol production strain to address a key question regarding the sensitivity of cells to short chain alcohols. In the past, we have defined specific point mutations in the translation initiation factor eIF2B based upon phenotypic resistance/sensitivity to high concentrations of exogenously added n-butanol. Here, we show that even during endogenous butanol production, a butanol resistant strain generates more butanol than a butanol sensitive strain.

**Conclusion:**

These studies demonstrate that appreciable levels of n-butanol can be achieved in *S. cerevisiae* but that significant metabolic manipulation is required outside of the pathway converting acetyl-CoA to butanol. Furthermore, this work shows that the regulation of protein synthesis by short chain alcohols in yeast is a critical consideration if higher yields of these alcohols are to be attained.

**Electronic supplementary material:**

The online version of this article (doi:10.1186/s13068-015-0281-4) contains supplementary material, which is available to authorized users.

## Background

Since fossil fuels represent a finite resource and their continued use contributes to climate change, alternative sources of energy have been widely sought [1]. Biofuels produced from fermentation of renewable resources are expected to represent an important replacement for gasoline [2]. Commercial bioethanol production from high yielding fermentations of the yeast *Saccharomyces cerevisiae* relies upon the inherent resistance of yeast cells to the damaging properties of ethanol [3]. However, ethanol’s low energy content and high hygroscopicity are viewed as disadvantages in terms of its quality as a fuel [4, 5]. n-Butanol (1-butanol) and other short chain alcohols have a range of physical properties, which make them superior fuels to ethanol [4]. For instance, in comparison to ethanol, n-butanol is less hygroscopic making it less corrosive, and it has a higher energy density and octane value. These characteristics mean that n-butanol can be mixed with gasoline in almost any proportion [4].

**Fig. 1:**
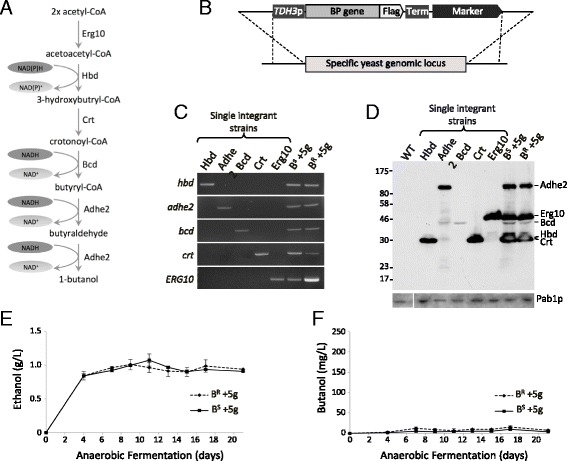
The ABE butanol pathway does not lead to high levels of butanol production in *S. cerevisiae*. **a** Schematic diagram of a butanol production pathway utilised by a variety of clostridial species as part of ABE fermentation. The Hbd (3-hydroxybutyryl-CoA dehydrogenase), Crt (3-hydroxybutyryl-CoA dehydratase), Bcd (butyryl-CoA dehydrogenase) and Adhe2 (alcohol dehydrogenase) enzyme genes were derived from *Clostridium beijerinckii*, and the Erg10 (thiolase) sequence was taken from *S. cerevisiae*. **b** The strategy for expression of these genes via genomic integration into *S. cerevisiae* is depicted. Codon-optimised cassettes bearing C-terminal Flag epitope tags were expressed from the strong *TDH3* gene promoter and *CYC1* terminator sequences. Each cassette also carries a different marker downstream and was integrated at a precise location associated with high level expression (see Methods). **c** PCR analysis on genomic DNAs derived from either single integrant strains or a strain that has been back-crossed such that it harbours all five cassettes. The primers used are specific to the genomic integration loci and the cassettes labelled to the left of the gel pictures. **d** Western blotting using an anti-Flag antibody to detect the expressed proteins in either the single integrant strains or the strains bearing all five cassettes. Protein products are labelled to the right of the gel image. A blot probed with an anti-Pab1p antibody provides a loading control (*lower panel*). **e** and **f** Graphs depicting the level of ethanol or butanol produced from butanol sensitive (*GCD1-S180*) or butanol resistant (*GCD1-P180*) strains bearing the five butanol production genes (B^S^ + 5 g or B^R^ + 5 g) over a 21-day anaerobic fermentation. Error bars are ± SEM from five biological repeats

Post World War I, n-butanol was produced from acetone-butanol-ethanol (ABE) clostridial fermentations [6]. Butanol production via this route (Fig. 1a) involves the intracellular conversion of acetyl-CoA derived from carbohydrate catabolism through a series of five enzymatic reactions to n-butanol. More specifically, thiolase catalyses a Claisen condensation reaction between two acetyl-CoA molecules producing acetoacetyl-CoA, which is then sequentially reduced through 3-hydroxybutyryl-CoA, crotonyl-CoA and butyryl-CoA to n-butanol [7]. Increasing commercial competition with fossil fuel-derived n-butanol supplanted this technology for largely economic reasons, although with respect to biofuel production it has renewed significance [7]. However, there are a number of problems that are associated with this n-butanol production route at the industrial scale. For instance, these can include product inhibition, the potential for bacteriophage contamination, sporulation during solventogenesis, the complicated two-stage multi-temperature fermentation reaction and the mixed fermentation products [5, 8]. Based upon these difficulties, a number of studies have attempted to produce n-butanol in other organisms. For instance, investigators have used both of the biotechnology workhorse model organisms, *Escherichia coli* and *S. cerevisiae* [9, 10].

Engineered *E. coli* bearing the ABE pathway have been generated in a number of different ways and have been shown to produce high levels of butanol [11, 12]. However, as for *Clostridia*, some problems still exist in the use of engineered *E. coli* for butanol production, including the potential for phage infection/fermentation spoilage and product/degradation product toxicity [13]. As *S. cerevisiae* is currently widely used for the production of bioethanol, it holds significant advantages in terms of scalable industrial fermentation for the production of butanol [14]. However, initial attempts at introducing the ABE pathway into *S. cerevisiae* produced very low yields of 2.5 mg/L [15]. Subsequent studies have generated improved yields by targeting specific metabolic pathways or utilising specific starting substrates [16, 17]. In addition, alternative pathways for butanol production have been sought with varying degrees of success [18, 19]. Recurrent issues associated with these butanol fermentations are relatively low yields and the potential for end-product toxicity.

Previously, we have studied, at the molecular level, mechanisms underlying the toxic effects of n-butanol and other alcohols in yeast [20–23]. We have found that these alcohols specifically inhibit protein synthesis at the translation initiation step by perturbing the guanine nucleotide exchange factor, eukaryotic initiation factor (eIF)2B [20, 21]. This factor recycles, eIF2, a key g-protein involved in translation initiation. eIF2 in the GTP bound form recruits the initiator methionyl tRNA to the ribosome [24]. As a consequence of translation initiation, GTP is hydrolysed on eIF2 generating eIF2-GDP, which requires eIF2B-dependent recycling before further rounds of translation initiation are possible.

In this study, we explore the hypothesis that yeast strains, which are more resistant to the toxic effects of n-butanol and other alcohols, are capable of producing more alcohol. In order to assess this question, we generated strains bearing the entire ABE pathway, as well as specific metabolic mutations designed to increase carbon flux towards the ABE pathway. As a result, we obtained a strain of yeast that is capable of producing up to 300 mg/L n-butanol. Overall, even though this level of n-butanol does not begin to approach the level required to inhibit eIF2B and generate toxicity, we observe a significant difference in the level of n-butanol produced in strains that only vary in their sensitivity/resistance to alcohols. Therefore, the toxicity of alcohols on cells is a significant factor when considering biofuel production and strategies aimed at overcoming this toxicity hold significant promise in the quest towards commercially economic biofuel yields.

## Results and discussion

### Addition of the ABE pathway to *S. cerevisiae* results in very low levels of n-butanol

The goal of this project at the outset was to determine whether the toxic effects of alcohols such as n-butanol are important in determining the yield from producing strains. We started with two parent strains that are isogenic apart from a point mutation in a gene encoding a translation initiation factor; *GCD1. GCD1-P180* (denoted B^R^ throughout) is resistant to 1 % butanol, whereas *GCD1-S180* (denoted B^S^ throughout) is sensitive to this level of exogenously added butanol. In order to evaluate this question, we generated B^S^ and B^R^ strains of yeast expressing four *Clostridia beijerinckii* genes and one yeast gene that together encode the enzymes of an ABE pathway. Previous studies had shown that yeast strains harbouring the genes for these enzymes on extremely high copy plasmids produced n-butanol at quite low levels of ~2.5 mg/L [15]. Therefore, we decided to integrate codon-optimised genes directly into specific sites associated with high expression on the genome [25] under the control of a highly efficient ubiquitous yeast *TDH3* gene promoter with a *CYC1* 3′ end formation sequence downstream. Each open reading frame (ORF) was also tagged with Flag epitopes to aid protein detection (Fig. 1b).

Individual genes were integrated into opposing mating type haploid yeast strains, such that via a combination of genetic crosses (see Methods), strains were constructed bearing all five genes (Fig. 1c). Western blotting using an anti-Flag monoclonal antibody confirmed that proteins of an appropriate size were expressed (Fig. 1d). However, when butanol was quantified from the strains under a variety of conditions, including anaerobic fermentation, very little butanol was recovered in the media (<10 mg/L) and levels of ethanol production were equivalent to the parent strains (Fig. 1e, f). The low butanol production observed in this strain was entirely consistent with previous attempts to produce n-butanol in *S. cerevisiae* [15].

### Deletion of the *ADH1* gene improves the n-butanol yield significantly

**Fig. 2:**
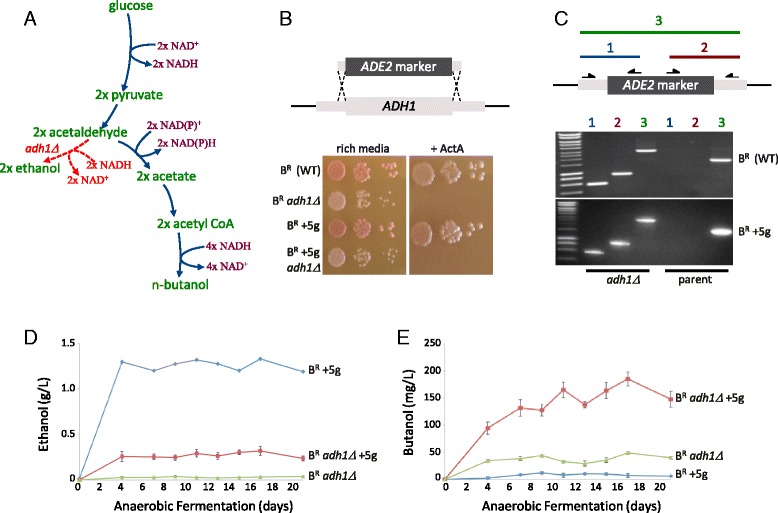
Deletion of the *ADH1* gene improves butanol production in *S. cerevisiae*. **a** A schematic diagram of how the pathway of glucose fermentation to ethanol is connected to the added butanol production pathway. The step affected by the *ADH1* deletion is highlighted and the balance of reducing equivalent in the form of NADH or NADPH through the pathway is detailed. **b** The strategy for *ADH1* deletion and screening of candidates on Actinomycin A plates. **c** PCR analysis on genomic DNAs derived from either the *adh1Δ* strains or their parent. The primers used and resulting PCR products are detailed above the gels. **d** and **e** Graphs depicting the level of ethanol or butanol produced from the *adh1Δ* strain or from strains bearing the five butanol production genes either alone (B^R^ + 5 g) or in combination with *adh1Δ* (B^R^ *adh1Δ* +5 g) over a 21-day anaerobic fermentation. Error bars are ± SEM from five biological repeats

A number of factors could be contributing to the poor butanol yields and we explored these in the B^R^ strain background. For instance, it is likely that a redox imbalance exists due to the high NADH requirements of the butanol production pathway, plus it is possible that the substrate for the butanol production pathway, cytosolic acetyl-CoA, is limiting. In an attempt to overcome these problems, we deleted the major yeast alcohol dehydrogenase gene *ADH1*. We reasoned that this deletion should improve the levels of NADH, as the enzyme is the primary route in yeast for balancing the NAD+ consumed by the glyceraldehyde 3-phosphate dehydrogenase step of glycolysis. In addition, deletion of *ADH1* could potentially increase cytosolic acetyl-CoA by causing the accumulation of acetaldehyde (Fig. 2a).

Therefore, a strategy was designed whereby the *ADH1* gene was deleted (Fig. 2b) to give strains with the previously described [26] actinomycin A sensitive phenotype (Fig. 2c). The *ADH1* deletion was subsequently confirmed by PCR on genomic DNA from the selected transformants (Fig. 2d).

Consistent with the deletion of a major alcohol dehydrogenase, growth and the levels of ethanol produced by the *adh1Δ* strain were very low compared to the wild type strain under anaerobic conditions (Fig. 2d and Additional file 1: Figure S1). In addition, glucose present at the outset was not entirely consumed during the fermentation (Additional file 1: Figure S1). Interestingly, for the strain bearing the butanol production pathway (B^R^ +5 g), *adh1Δ* still reduced ethanol levels dramatically but not to the same extent as an *adh1Δ* strain lacking the butanol pathway (Fig. 2d). Furthermore, the impact of deleting the *ADH1* gene in this strain was less pronounced in terms of growth and glucose consumption (Additional file 1: Figure S1). It is possible these minor fermentation improvements stem from the fact that the clostridial Adhe2 alcohol dehydrogenase is expressed as part of the butanol pathway, and this enzyme might to a small extent rescue production of ethanol from acetaldehyde.

Intriguingly, deletion of *ADH1* also leads to the production of n-butanol. Recent studies suggest that in the absence of *ADH1*, an endogenous pathway of n-butanol production can be activated [18]. This pathway likely stems from threonine catabolism [18] and appears to be responsible for the production of roughly 40 mg/L n-butanol from our strain (Fig. 2e). However, when an *adh1Δ* mutant is generated in the context of the strain harbouring the butanol production pathway (B^R^*adh1Δ* +5 g), approximately 150 mg/L n-butanol is generated (Fig. 2e).

**Fig. 3:**
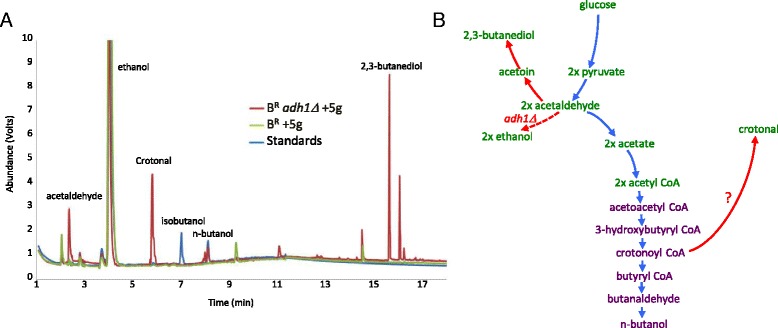
Deletion of *ADH1* in strains with the butanol production pathway leads to accumulation of side-pathway intermediates. **a** A Gas-chromatograph from a GC-MS analysis of media from the B^R^ + 5 g (*green*) and *adh1Δ* B^R^ + 5 g (*red*) yeast strains. Standards of butanol, isobutanol and ethanol were also run and are shown for comparison (*blue*). Specific peaks where a compound was identified by mass spectrometry are labelled. **b** A schematic diagram of the pathway of glucose fermentation to ethanol connected to the added butanol production pathway with potential side pathways activated in an *adh1Δ* mutant shown in red

In order to explore the profile of chemicals produced by these strains of yeast, a gas chromatography-mass spectrometry (GC-MS) analysis was undertaken (Fig. 3a). This revealed that for strains bearing the butanol production enzymes, deletion of *ADH1* led to the appearance of a number of new peaks on the gas chromatograph. Mass spectrometry revealed likely identities for many of these peaks, which were explicable in terms of the metabolism of yeast. For instance, the accumulation of a peak corresponding to acetaldehyde and reduced levels of ethanol is entirely consistent with the removal of a major alcohol dehydrogenase. Furthermore, the production of 2,3-butanediol from acetaldehyde likely represents a means to restore the redox imbalance caused by removal of this major alcohol dehydrogenase. Finally, the accumulation of a peak identified as crotonal is intriguing. It is entirely possible that this derives from crotonyl-CoA via the action of a broad specificity aldehyde reductase in yeast. Overall, our interpretation of these data is that the result of the *adh1Δ* is an accumulation of acetaldehyde, which results in increased levels of 2,3-butanediol. Hence, the production of acetyl-CoA from acetaldehyde is not a favoured route as would be required for maximal butanol production. However, improved levels of butanol are being attained and it appears that intermediates in the butanol pathway or derivatives of them such as crotonal may be accumulating (Fig. 3b).

### Replacement of the Bcd gene with Ter does not significantly improve butanol yields

**Fig. 4:**
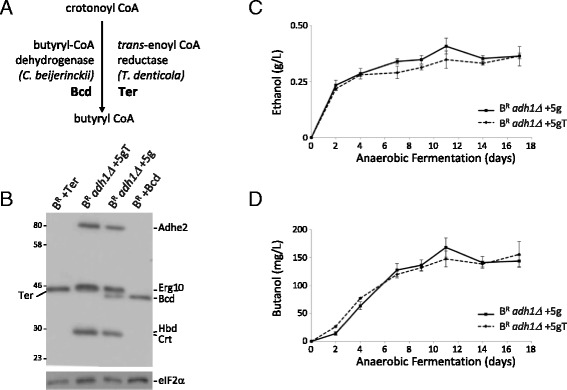
Replacement of butyryl-CoA dehydrogenase (Bcd) with *trans*-enoyl-CoA reductase (Ter) does not substantially improve butanol levels. **a** A schematic showing the reaction involved and the replacement strategy. **b** Western blotting using an anti-Flag antibody to detect the expressed proteins in extracts from *adh1* mutant strains bearing the butanol production pathway with Bcd (B^R^ *adh1Δ* +5 g) or with Ter (B^R^ *adh1Δ* +5gT) relative to extracts from control strains bearing just Bcd (B^R^ + Bcd) or Ter (B^R^ + Ter). Protein products are labelled to the *right* and *left* of the gel image. A blot probed with an anti-eIF2α antibody provides a loading control (*lower panel*). **c** and **d** Graphs depicting the level of ethanol or butanol produced from *adh1Δ* mutant strains bearing five butanol production genes with either Bcd (B^R^ *adh1Δ* +5 g) or Ter (B^R^ *adh1Δ* +5gT) over a 21-day anaerobic fermentation. Error bars are ± SEM from five biological repeats

On the basis of the GC-MS data above, a number of discrete strategies were attempted to improve butanol yields further. The first strategy revolved around the accumulation of crotonal as a possible derivative of crotonyl-CoA. This suggests that the Bcd enzyme in the butanol production pathway maybe be somehow deficient. Intriguingly, the levels of the Bcd protein were the lowest of the five added proteins when assessed by western blotting (Fig. 1d). During studies on butanol synthesis in *E. coli* [11, 12], an alternative non-flavin dependent enzyme has been described as a more effective alternative to Bcd: a *trans*-enoyl-CoA reductase (Ter) enzyme from *Treponema denticola* (Fig. 4a). Therefore, a strategy was undertaken to test whether the replacement of Bcd with Ter led to improvements in butanol levels.

A codon-optimised ORF for the Ter gene was used to precisely supplant the Bcd ORF in the integration cassette, and therefore, a directly comparable Ter containing strain was obtained (Fig. 4). In contrast to what has been observed in *E. coli* [11, 12] and even though the levels of Ter were as high as the other integrated genes of the butanol pathway (Fig. 4b), the presence of the Ter gene did not alter the level of ethanol (Fig. 4c) or lead to significant improvements in the butanol titre (Fig. 4d).

### Improved flux of carbon to acetyl CoA generates higher butanol levels

**Fig. 5:**
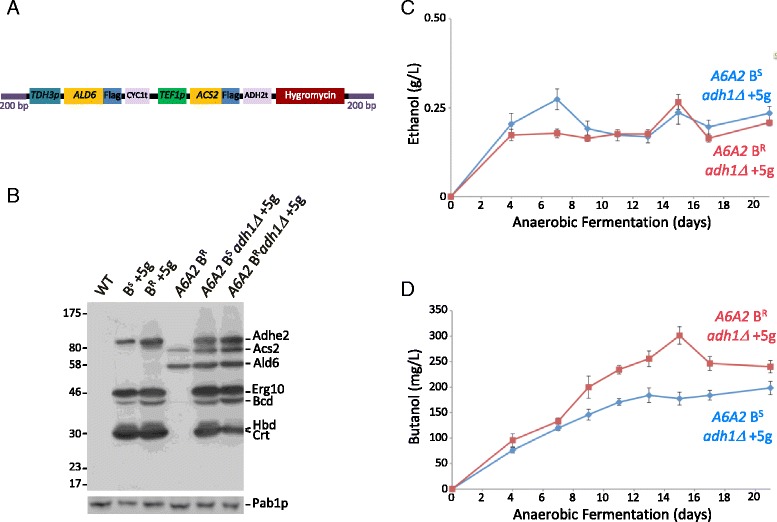
Expression of an acetyl-CoA driving force in the context of a butanol resistant allele of *GCD1* further improves butanol levels. **a** A schematic diagram of the yeast genomic integration cassette designed to drive high level *ALD6 ACS2* expression. **b** Western blotting using an anti-Flag antibody on protein samples from strains where the *ALD6 ACS2* (*A6A2*) cassette has been integrated relative to the parent strains bearing the five butanol production enzymes and controls. Protein products are labelled to the *right* of the gel image. A blot probed with an anti-Pab1p antibody provides a loading control (*lower panel*). **c** and **d** Graphs depicting the level of ethanol or butanol produced from *adh1Δ* mutant strains bearing five butanol production genes (+5 g) and the *ALD6 ACS2* expression cassette (*A6A2*). Data from 21-day anaerobic fermentations for both B^R^ (*GCD1-P180*) and B^S^ (*GCD1-S180*) derived strains are shown. Error bars are ± SEM from six biological repeats

The accumulation of acetaldehyde, acetate and 2,3-butanediol in the GC-MS analysis for the *adh1Δ* strains bearing the butanol production pathway is suggestive that the flux towards the butanol pathway is not in any way maximal. The enzymes involved in the conversion of acetaldehyde to acetyl-CoA are the Ald6p cytosolic aldehyde dehydrogenase and the acetyl-CoA synthase Acs2p. The expression of these genes is carefully controlled and predominantly induced where non-fermentable carbon sources are being metabolised or under stress conditions [27]. Therefore, to obviate this regulation, we decided to express the *ALD6* and *ACS2* genes from highly active constitutive promoters in the *adh1Δ* mutant bearing the ABE pathway. An integration cassette was designed (Fig. 5a) where the expression of *ALD6* was placed under the control of the *TDH3* promoter with a *CYC1* 3′ end formation sequence, while *ACS2* was expressed from the *TEF1* promoter with *ADH1* 3′ end processing signals. Both ORFs were codon-optimised and Flag-tagged at the C-terminus to allow expression to be monitored relative to the other enzymes of the butanol production pathway. Here, expression of all seven transgenes in the strain was found to be roughly comparable (Fig. 5b). Even though expression of both Ald6p and Ald2p was observed, little difference was noted in the levels of acetaldehyde and crotonal produced on GC-MS traces (Additional file 2: Figure S2). However, evaluation of the resulting strain in terms of butanol and ethanol production showed that expression of Ald6p and Acs2p gave a small improvement in peak butanol levels from 150–175 mg/L (Figs. 2d and 3d) to 250-300 mg/L (Fig. 5d). This is consistent with other studies where improvements in cytosolic acetyl-CoA availability gave small increases in butanol yields [17].

### Butanol resistant strains generate higher levels of butanol

Having generated a strain that yields a reasonable level of butanol, we assessed the impact of butanol resistance/sensitivity at the level of translation initiation. Previous work from the lab has defined specific butanol resistance and sensitive mutations in the genes for eIF2B. In this case, we generated strains, which harboured allelic variation at the *GCD1* locus, which encodes the γ subunit of eIF2B. A proline at residue 180 gives a resistant phenotype, whereas a Serine at this position increases sensitivity to butanol.

The resulting strains were tested for alcohol production using our standard assay system, and the butanol resistant strain reproducibly generated up to 1.5–2-fold higher peak levels of butanol (Fig. 5d). These results were unforeseen, as the level of butanol generated by these strains is significantly lower than the level added exogenously during the tolerance studies [20, 21]. In addition, the level of ethanol production was slightly reduced at early time points in the butanol resistant strain (Fig. 5c). This is suggestive that in the butanol resistant strain, a higher flux is attained towards butanol and away from ethanol than in the butanol sensitive strain. These results provide proof of principle that strains that are more resistant to the effects of butanol (and other fusel alcohols) have improved yields of these alcohols from production pathways.

## Conclusions

In this study, we show that an exogenous ABE pathway only generates substantial levels of butanol in yeast when a number of metabolic alterations are made. Deletion of the major alcohol dehydrogenase *ADH1* not only leads to butanol production via a previously described endogenous pathway but also promotes much higher levels of butanol where an exogenous butanol production pathway has been added. These data support a view that both the endogenous and exogenous pathways are active in the cells.

Our GC-MS studies highlight a number of potential bottlenecks particularly with regard to the exogenous pathway. Accumulation of crotonal led us to take an approach previously validated in *E. coli*: the replacement of the Bcd enzyme with Ter [11, 12]. However in our studies in yeast, Ter gives little improvement in butanol levels. It seems possible that neither of these enzymes is particularly efficient in the context of the yeast cytoplasm, and this could represent an area where substantial further improvements in yield are possible. The GC-MS data also show that acetaldehyde, acetate and 2,3-butanediol accumulate in an *adh1Δ* mutant bearing the ABE pathway. The accumulation of these compounds suggests that production of cytosolic acetyl-CoA from acetaldehyde occurs inefficiently. Therefore, a high expression strategy was applied to the *ALD6* and ACS2 genes involved in this process. In strains, this metabolic alteration generated a moderate improvement in the levels of butanol from the strain; peak levels increase from ~175 to ~300 mg/L. Therefore, while stimulating cytosolic acetyl-CoA production does lead to an improvement in butanol production, a deficiency in this area is not a major limitation. This begs the question what is the major limitation that prevents greater butanol production. Possible answers lie in an imbalance in redox potential or in sensitivity of the cells to butanol itself or intermediates in the pathway.

The initial goal of this project was to assess whether differences in the sensitivity of strains to butanol prompted equivalent changes in the yield of butanol. Here, we use previously characterised strains bearing butanol sensitive and butanol resistant alleles of the *GCD1* gene to provide proof of principle that the inherent sensitivity of yeast strains to butanol impacts upon butanol production. Given that the concentrations of butanol that are required to inhibit protein synthesis and growth (1–2 %, 10–20 g/L) are very different to the levels that are produced in our yeast strains (0.3 g/L), it is inherently quite startling that greater levels of butanol are produced in a butanol resistant strain. Our current working hypothesis to explain this discrepancy is that butanol transport across the yeast cell membrane is inefficient. Thus, if extracellular butanol does not pass into a cell and intracellular butanol does not pass out of a cell particularly well, it is possible that the level of extracellular butanol required to inhibit growth and translation would be high, whereas the level of endogenous butanol required to elicit the same effect could be much lower. Indeed, a role for specific efflux pumps in increasing the tolerance of *E. coli* to exogenously added short chain alcohols has been described [28]. This opens up the possibility of an integrated approach towards improved tolerance to, and hence, improved production of, short chain alcohols in *S. cerevisiae*, where both intracellular resistance at the level of proteins synthesis and the cells capacity to export alcohols are enhanced.

## Methods

### Yeast growth and strain construction/validation

Strains used in this study were grown at 30 °C on either standard yeast extract/peptone/dextrose media (YPD) or synthetic complete dextrose media (SCD) both supplemented with 2 % glucose [29]. Individual genomic integration and deletion cassettes were generated and transformed into yeast using standard PCR-based integration methods to target the integration cassettes to specific high expression sites in the yeast genome [25] and validated using PCR, western blotting and phenotypic analysis. The individual cassettes carried yeast codon-optimised sequences Ter (from *T. denticola*), Cct, Adhe2, Bcd and Hbd (from *Clostridium Beijerinckii*) with a C-terminal Flag tag (two Flag peptide epitopes) and the *CYC1* terminator sequences downstream. Each gene was first inserted into a specific pRS vector with a *TDH3* promoter inserted upstream and the auxotrophic marker gene immediately downstream of the cassette. Integration primers were then designed to isolate the cassette upstream of the *TDH3* promoter to downstream of the auxotrophic marker (Fig. 1b). The sites of integration were selected based on previous studies analysing the efficiency of gene expression from various sites across the yeast genome. The *ADH1* gene was deleted using the *ADE2* marker using standard yeast PCR-based gene disruption methods. The codon-optimised *ERG10* yeast gene was synthesised downstream of the *TDH3* gene promoter and upstream of the *CYC1* terminator sequence, and flag epitope tags were placed at the C-terminus. The cassette was sub-cloned into the pFa6-KanMX4 plasmid upstream of the *KanMX4* gene. Integration primers were designed to amplify the entire fragment prior to transformation into yeast. Codon-optimised versions of the yeast *ALD6* and *ACS2* genes were synthesised downstream of the *TDH3* and *TEF1* gene promoters and upstream of the *CYC1* and *ADH2* terminator sequences, respectively. Flag epitope tags were placed at the C-terminus of each cassette and a hygromycin marker gene was added (Fig. 5a). The whole cassette was bounded by 200 n sequences directing it to the *TRP1* locus in the yeast genome. Finally, the cassette was flanked by sites for the type IIS restriction enzyme, *Bsp*QI, such that the whole fragment could be released and transformed into yeast. All commercial DNA synthesis was carried out by either Mr Gene GmbH (Regensburg, Germany) or GenScript (Piscataway, NJ).

### Measurements of butanol and ethanol

Strains were grown in liquid YPD media from a starting OD_600_ of 0.1 using semi-anaerobic 50 ml vials over a 21-day period. On specific days, 2 ml samples were taken, passed through a 0.22 μ filter into gas chromatography (GC) vials and analysed by GC-FID using an Agilent 6850A GC system with an Agilent 4513A automatic injector, sampler and controller (Agilent technologies Ltd., Stockport, UK). A J&W DB-WAX capillary column (30 m × 0.25 mm, 0.25 μM) (Agilent technologies Ltd.) was used for separation. Samples were quantified relative to standards of ethanol and butanol.

GC-MS was carried out using media from anaerobic cultures grown in YPD for 5 days. Using a 6890 N GC system coupled to a 5973 Mass Selective Detector (MSD) (Agilent technologies Ltd.), 2 ml samples were collected and analysed. Data was analysed and processed using the MSD ChemStation software (Agilent technologies Ltd.).

### Western blot analysis of Flag-tagged proteins

Yeast culture (5 ml) were grown to an OD_600_ of 0.7 in YPD, pelleted; then protein samples were prepared and processed for electrophoresis and immunoblot analysis as described previously [30]. A monoclonal anti-Flag antibody (Sigma-Aldrich, Dorset, UK) was used as the primary antibody for the detection.

## Additional files

Additional file 1: Figure S1. Growth (OD_600_) and glucose consumption (%) for the strains indicated over 21 day anaerobic fermentations. Error bars are ± SEM from 3 biological repeats. Additional file 2: Figure S2. A Gas chromatograph from a GC-MS analysis of media from the *A6A2* B^R^ *adh1Δ* 5 g (blue) yeast strains relative to standards of butanol, isobutanol and ethanol (red). Specific peaks where a compound was identified by mass spectrometry are labelled.
