# Supplementary material for: Butanol production in S. cerevisiae via a synthetic ABE pathway is enhanced by specific metabolic engineering and butanol resistance
Source: Biotechnol Biofuels. 2015 Jul 8;8:97. doi: 10.1186/s13068-015-0281-4 (PMC4501090; doi:10.1186/s13068-015-0281-4)
Supplement: Additional file 1: Figure S1. — Growth (OD600) and glucose consumption (%) for the strains indicated over 21 day anaerobic fermentations. Error bars are ± SEM from 3 biological repeats. [file 13068_2015_281_MOESM1_ESM.pptx]

## Slide 1
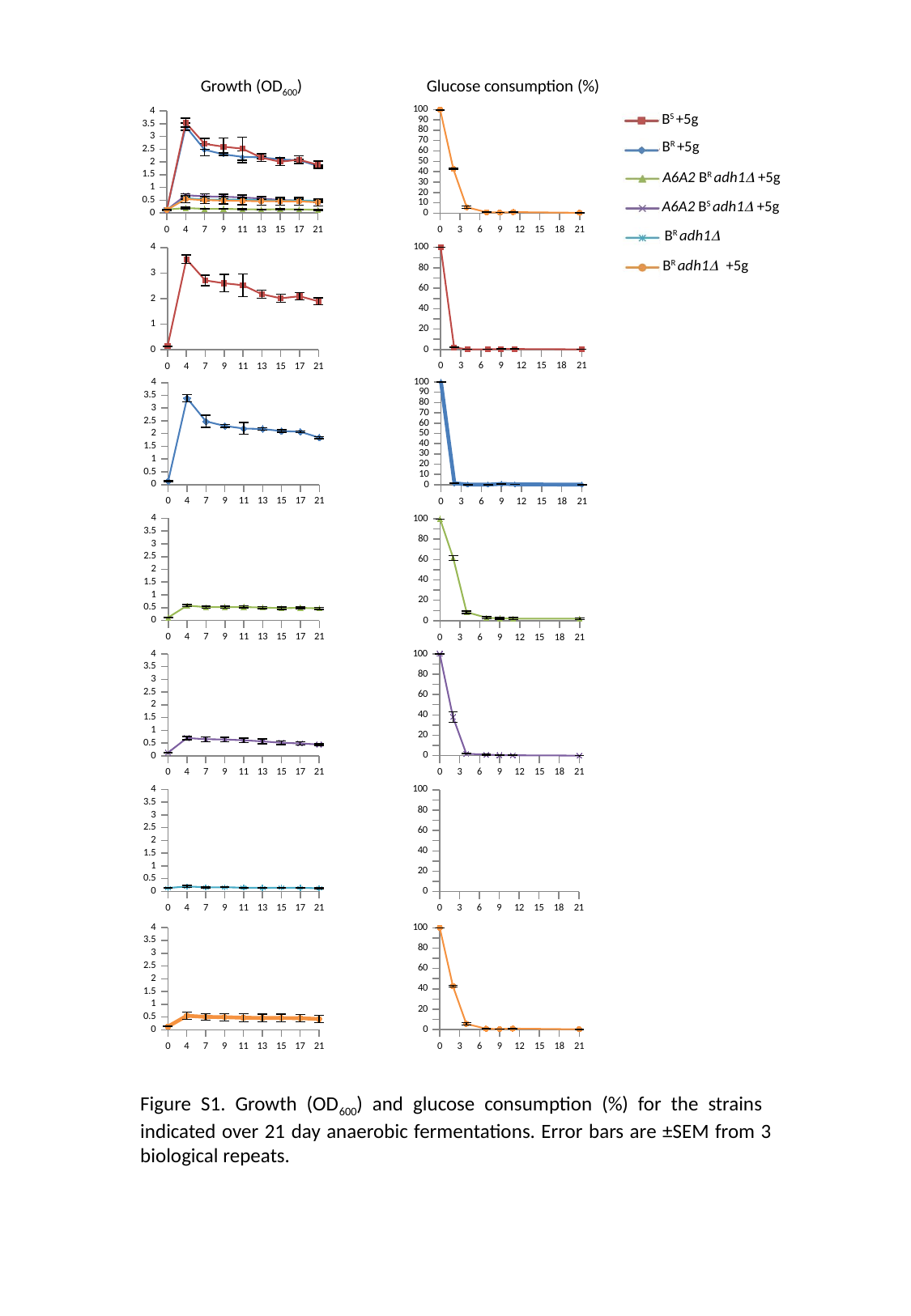

Growth (OD600)
Glucose consumption (%)
### Chart
| Category | BUTR+5g | BUT S+ 5g | adh1.ALD6.ACS2.BUTR+5g | adh1.ALD6.ACS2.BUTS+5g | adh1.ymk23 | adh1.BUTR+5g |
|---|---|---|---|---|---|---|
### Chart
| Category | BUTR+5g | BUT S+ 5g | adh1.ymk23 | adh1.BUTR+5g | adh1.ALD6.ACS2.BUTR+5g | adh1.ALD6.ACS2.BUTS+5g |
|---|---|---|---|---|---|---|
| 0.0 | 0.136 | 0.127 | 0.131333333333333 | 0.12 | 0.116666666666667 | 0.122666666666667 |
| 4.0 | 3.39 | 3.54 | 0.193333333333333 | 0.695333333333333 | 0.583666666666667 | 0.545666666666667 |
| 7.0 | 2.48 | 2.71 | 0.160666666666667 | 0.648666666666667 | 0.520333333333333 | 0.500666666666667 |
| 9.0 | 2.3 | 2.6 | 0.160333333333333 | 0.638 | 0.522 | 0.489 |
| 11.0 | 2.2 | 2.52 | 0.147666666666667 | 0.607333333333333 | 0.527 | 0.468666666666667 |
| 13.0 | 2.18 | 2.17 | 0.136666666666667 | 0.565333333333333 | 0.504 | 0.459666666666667 |
| 15.0 | 2.1 | 2.01 | 0.142666666666667 | 0.51 | 0.481666666666667 | 0.457333333333333 |
| 17.0 | 2.07 | 2.09 | 0.139333333333333 | 0.484666666666667 | 0.492 | 0.453 |
| 21.0 | 1.84 | 1.89 | 0.122333333333333 | 0.440666666666667 | 0.471333333333333 | 0.418 |
BS +5g
BR +5g
A6A2 BR adh1D +5g
A6A2 BS adh1D +5g
BR adh1D
### Chart
| Category | BUT S+ 5g |
|---|---|
### Chart
| Category | BUT S+ 5g |
|---|---|
| 0.0 | 0.127 |
| 4.0 | 3.54 |
| 7.0 | 2.71 |
| 9.0 | 2.6 |
| 11.0 | 2.52 |
| 13.0 | 2.17 |
| 15.0 | 2.01 |
| 17.0 | 2.09 |
| 21.0 | 1.89 |BR adh1D +5g
### Chart
| Category | BUTR+5g |
|---|---|
| 0.0 | 0.136 |
| 4.0 | 3.39 |
| 7.0 | 2.48 |
| 9.0 | 2.3 |
| 11.0 | 2.2 |
| 13.0 | 2.18 |
| 15.0 | 2.1 |
| 17.0 | 2.07 |
| 21.0 | 1.84 |
### Chart
| Category | BUTR+5g |
|---|---|
### Chart
| Category | adh1.ALD6.ACS2.BUTR+5g |
|---|---|
### Chart
| Category | adh1.ALD6.ACS2.BUTR+5g |
|---|---|
| 0.0 | 0.116666666666667 |
| 4.0 | 0.583666666666667 |
| 7.0 | 0.520333333333333 |
| 9.0 | 0.522 |
| 11.0 | 0.527 |
| 13.0 | 0.504 |
| 15.0 | 0.481666666666667 |
| 17.0 | 0.492 |
| 21.0 | 0.471333333333333 |
### Chart
| Category | adh1.ALD6.ACS2.BUTS+5g |
|---|---|
### Chart
| Category | adh1.BUTR+5g |
|---|---|
| 0.0 | 0.12 |
| 4.0 | 0.695333333333333 |
| 7.0 | 0.648666666666667 |
| 9.0 | 0.638 |
| 11.0 | 0.607333333333333 |
| 13.0 | 0.565333333333333 |
| 15.0 | 0.51 |
| 17.0 | 0.484666666666667 |
| 21.0 | 0.440666666666667 |
### Chart
| Category | adh1.ymk23 |
|---|---|
### Chart
| Category | adh1.ymk23 |
|---|---|
| 0.0 | 0.131333333333333 |
| 4.0 | 0.193333333333333 |
| 7.0 | 0.160666666666667 |
| 9.0 | 0.160333333333333 |
| 11.0 | 0.147666666666667 |
| 13.0 | 0.136666666666667 |
| 15.0 | 0.142666666666667 |
| 17.0 | 0.139333333333333 |
| 21.0 | 0.122333333333333 |
### Chart
| Category | adh1.BUTR+5g |
|---|---|
### Chart
| Category | adh1.ALD6.ACS2.BUTS+5g |
|---|---|
| 0.0 | 0.122666666666667 |
| 4.0 | 0.545666666666667 |
| 7.0 | 0.500666666666667 |
| 9.0 | 0.489 |
| 11.0 | 0.468666666666667 |
| 13.0 | 0.459666666666667 |
| 15.0 | 0.457333333333333 |
| 17.0 | 0.453 |
| 21.0 | 0.418 |Figure S1. Growth (OD600) and glucose consumption (%) for the strains indicated over 21 day anaerobic fermentations. Error bars are ±SEM from 3 biological repeats.
