# Supplementary material for: Butanol production in S. cerevisiae via a synthetic ABE pathway is enhanced by specific metabolic engineering and butanol resistance
Source: Biotechnol Biofuels. 2015 Jul 8;8:97. doi: 10.1186/s13068-015-0281-4 (PMC4501090; doi:10.1186/s13068-015-0281-4)
Supplement: Additional file 2: Figure S2. — A Gas chromatograph from a GC-MS analysis of media from the A6A2 BR adh1Δ 5 g (blue) yeast strains relative to standards of butanol, isobutanol and ethanol (red). Specific peaks where a compound was identified by mass spectrometry are labelled. [file 13068_2015_281_MOESM2_ESM.ppt]

## Slide 1
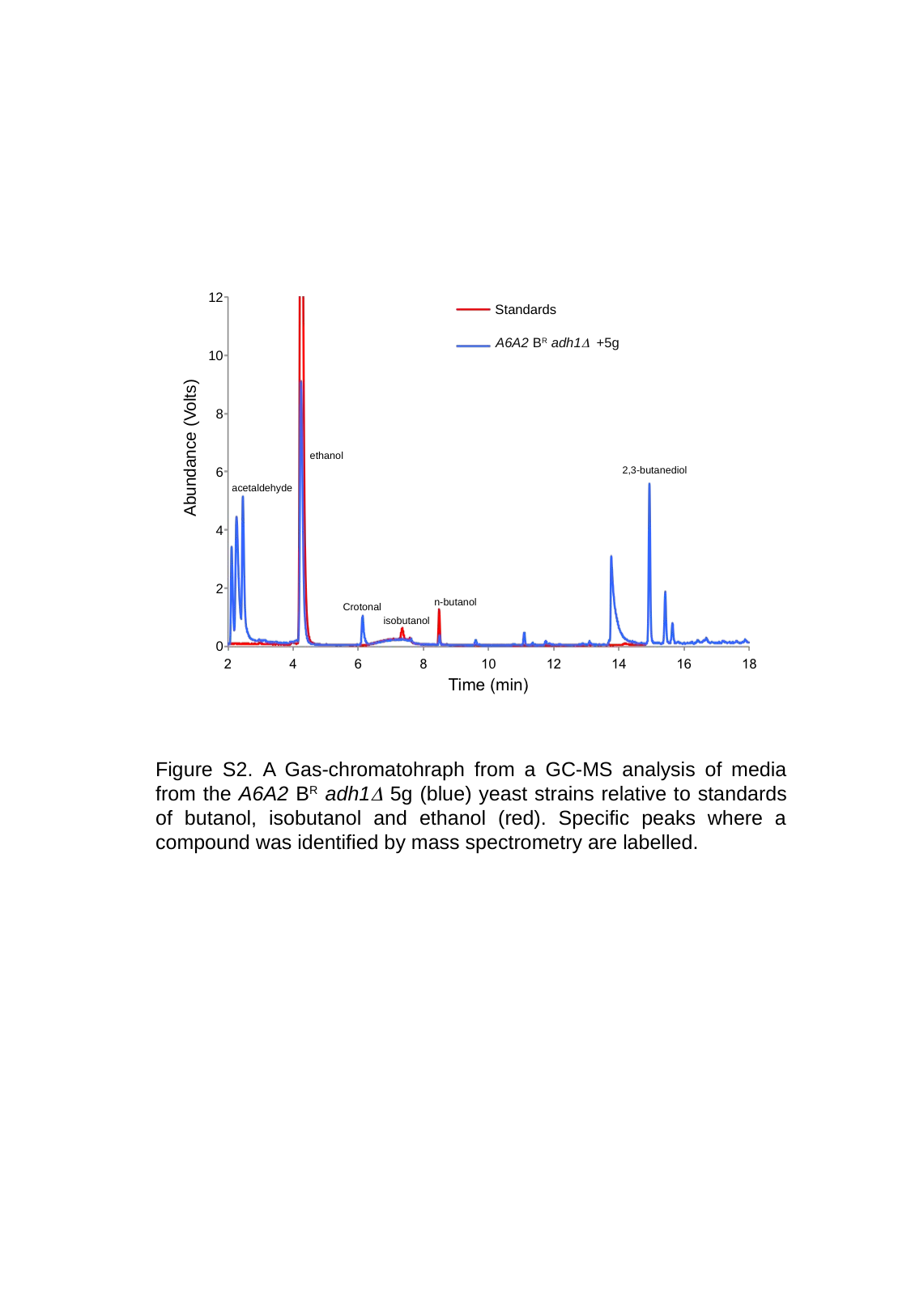

12
Standards
A6A2 BR adh1Δ +5g
10
8
Abundance (Volts)
ethanol
6
2,3-butanediol
acetaldehyde
4
2
n-butanol
Crotonal
isobutanol
0
Figure S2. A Gas-chromatohraph from a GC-MS analysis of media from the A6A2 BR adh1Δ 5g (blue) yeast strains relative to standards of butanol, isobutanol and ethanol (red). Specific peaks where a compound was identified by mass spectrometry are labelled.
